# A Label-Free G-Quadruplex/Thioflavin T Fluorescent Sensor for ClO^−^ Detection: Implications for Stress-Induced Hypertension Biomarker Analysis

**DOI:** 10.3390/bios16030169

**Published:** 2026-03-18

**Authors:** Jianting Liu, Yaru Zhao, Linfang Zhang, Haisheng Liu, Guosong Zhang

**Affiliations:** 1 School of Life Sciences and Medicine, Shandong University of Technology, Zibo 255000, China; 2 College of Agriculture and Bioengineering, Heze University, Heze 274015, China

**Keywords:** hypochlorite ion (ClO^−^), G-quadruplex, phosphorothioate (PS), Thioflavin T (ThT), stress-induced hypertension, biomarker

## Abstract

The objective of this study is to develop a label-free fluorescent sensor for the quantitative detection of hypochlorite ions (ClO^−^) and validate its applicability in biological samples, particularly exploring the potential of ClO^−^ as a biomarker for stress-induced hypertension (SIH). Male Sprague-Dawley rats (8 weeks old, 250–300 g) were used to establish the SIH model. A guanine-rich (G-rich) signal DNA sequence (S-DNA) was rationally designed, with a ClO^−^-responsive phosphorothioate (PS) moiety integrated into the probe architecture. In the absence of ClO^−^, the S-DNA folds into a stable G-quadruplex structure, which specifically binds to ThT and triggers a significant enhancement of the dye’s fluorescence intensity. Upon introduction of ClO^−^, the specific hydrolysis reaction between the PS moiety and ClO^−^ induces cleavage of the S-DNA into two discrete fragments, thereby abrogating G-quadruplex formation and resulting in a remarkable quenching of ThT fluorescence. This proposed method exhibits excellent anti-interference capability against other reactive oxygen species (ROS) and achieves a low detection limit of 41.2 nM for ClO^−^. Furthermore, this strategy was successfully applied to the quantitative determination of endogenous ClO^−^ in human cells and the plasma of stress-induced hypertensive (SIH) rats, highlighting its substantial potential for clinical and physiological research.

## 1. Introduction

Hypochlorous acid (HClO) is a key reactive oxygen species (ROS) in biological systems. Accumulating evidence has demonstrated that aberrantly elevated levels of ClO^−^ in organisms can trigger oxidative stress responses, which are closely associated with the pathogenesis of various diseases, including tissue damage, inflammation, cardiovascular disorders, neurodegenerative diseases, and cancer [1,2,3]. Consequently, the accurate and selective detection of ClO^−^ concentrations in biological matrices is of paramount significance for elucidating related physiological and pathological mechanisms.

To date, various analytical techniques have been established for ClO^−^ detection, such as colorimetry, electrochemical analysis, chemiluminescence, and chromatography [4,5,6]. Among these methods, fluorescent probes offer distinct advantages, including high sensitivity, excellent resolution, simple operation, and rapid response, making them promising tools for ClO^−^ sensing [7]. Until now, multiple fluorescent probes have been reported for ClO^−^ detection, each with its own advantages and drawbacks [8,9,10,11]. The main drawbacks of these fluorescence methods are complex synthetic methods and interference from other ROS [6,12,13,14]. Thus, the development of a rapid, efficient, and highly selective fluorescent method for ClO^−^ detection remains an urgent need.

Phosphorothioate (PS) is an oligonucleotide in which the oxygen atom normally linking two consecutive nucleotides is replaced by sulfur and which resists degradation by cellular enzymes [15]. Recent studies have revealed that the specific reaction between PS and ClO^−^ can trigger the cleavage of DNA backbones, resulting in DNA fragmentation [16,17,18]. Thioflavin T (ThT), a water-soluble benzothiazole dye, has been extensively utilized as a fluorescent reporter for G-quadruplex structures, as it can specifically bind to G-quadruplexes formed by G-rich oligonucleotides and exhibit a dramatic fluorescence enhancement [19]. Due to its low cost, minimal background signal, good water solubility, and ease of use, ThT-based fluorescent probes have gained widespread attention in biosensing applications [19,20]. Previously, we reported a turn-on sensing method for ClO^−^ based on a G-quadruplex/ThT platform [21]. However, the reliance on DNA hybridization makes its detection performance highly susceptible to temperature, pH, and ionic strength, which interfere with strand displacement and G-quadruplex formation and thus limit practical applications. To overcome these limitations, we herein develop a rapid, label-free, and hybridization-free fluorescent strategy for the quantitative detection of ClO^−^. By ingeniously integrating ThT-triggered G-quadruplex formation with the specific oxidative cleavage of PS-modified DNA toward ClO^−^, the proposed platform avoids the tedious and environment-sensitive DNA hybridization process, thus featuring improved robustness, faster response, and greater potential for real-sample analysis. To test if the sensor works in biological samples and if ClO^−^ can be an SIH biomarker, we used male SD rats. These rats a reliable hypertension model with well-understood cardiovascular systems, similar to humans in oxidative stress and blood pressure control. This means our findings from rats can help understand ClO^−^’s role in human SIH.

## 2. Experimental Section

### 2.1. Materials and Reagents

The G-rich signal DNA sequence (S-DNA): 5′-GGGTAGGG*CGGGTTGG-3′, (* represents the phosphorothioate linkage) was synthesized by Sangon Biotechnology Co., Ltd. (Shanghai, China). NaClO was purchased from Macklin Reagent (Shanghai, China). Thioflavin T (ThT) was purchased from Sigma-Aldrich (St. Louis, MO, USA). All other reagents were of analytical-reagent grade. Ultra-pure water (18.25 MΩ·cm^−1^) was used in all experiments.

### 2.2. Apparatus

All fluorescence measurements were carried out on the INFINITEEPLEX Multifunctional Microplate Reader (Tecan Austria GmbH, Grödig, Austria). ThT was selected as the fluorescent dye, with an excitation wavelength of 425 nm and an optimal emission wavelength of 490 nm. For sample fluorescence detection, the excitation wavelength was set at 425 nm, and the emission wavelength range was 450–600 nm.

### 2.3. Reaction Condition Optimization

To achieve optimal detection performance, key experimental parameters, including S-DNA concentration and ThT concentration, were optimized. The fluorescence quenching efficiency (Q%) was used as the evaluation index, calculated according to the following equation:

$$Q\%=\frac{F0-F}{F0}\times 100\%$$

where F0 represents the fluorescence intensity in the absence of ClO^−^, and F denotes the fluorescence intensity after the addition of ClO^−^.

### 2.4. Quantitative Analysis of ClO^−^

Under the optimized conditions, quantitative analysis of ClO^−^ was performed. The reaction system (100 μL) consisted of 20 mM Tris-HCl buffer (pH 7.5), 500 nM S-DNA, 20 μM ThT, and various concentrations of ClO^−^ (0, 0.05, 0.1, 0.25, 0.5, 1, 2.5, 5, 10, and 25 μM). After incubation at room temperature for 5 min, fluorescence measurements were carried out.

### 2.5. Determination of Intracellular ClO^−^ Level in Human Cells

Human colorectal cancer cell line HCT-15 (from ATCC) were cultured in RPMI-1640 medium containing 10% FBS and penicillin-streptomycin at 37 °C in a humidified atmosphere with 5% CO_2_. After 24 h of incubation, ~1 × 10^6^ cells were collected, and the cell suspension was centrifuged at 2000× *g* at 4 °C for 10 min. The supernatant was discarded, and the cell pellets were lysed with 100 μL RIPA lysis buffer. Under optimized conditions, intracellular ClO^−^ content was quantified by fluorescence assay. Briefly, the reaction mixture was prepared with 100 μL of 1× reaction buffer (20 mM Tris-HCl, pH 7.5), 20 μM ThT, 500 nM S-DNA, and appropriate cell lysate. After 5 min incubation at room temperature, fluorescence signals were detected for ClO^−^ concentration calculation.

### 2.6. ClO^−^ Levels in Plasma of Rats with Stress-Induced Hypertension (SIH)

The SIH rat model was established following previously reported protocols [22]. Male Sprague-Dawley rats (8 weeks old, 250–300 g) were purchased from Jinan Pengyue Experimental Animal Co., Ltd. (Jinan, China). and individually housed in a pathogen-free facility at Heze University under controlled conditions (50–60% relative humidity, 12 h light/dark cycle, 23 ± 1 °C) with ad libitum food and water. The minimum number of animals was used to minimize distress. SIH models were established per previous protocols: rats were placed in 22 × 22 × 28 cm mesh-bottomed cages and exposed to unpredictable electric foot shocks (35–80 V, 100 ms pulse width, 2–30 s intervals) plus 88–98 dB buzzer noise (conditioned stimulus) for 2 h twice daily (9–11 am, 3–5 pm) for 15 days. Control rats were housed in identical cages without stress exposure. Femoral arterial blood pressure was monitored using the AD Instruments PowerLab system. Rats were anesthetized using pentobarbital sodium (60 mg/kg, i.p.), after which their abdominal cavities were opened, and blood was collected from the inferior vena cava into EDTA-containing blood tubes. The collected blood was centrifuged at 3000× *g* for 10 min to harvest the plasma. Under optimized experimental conditions, the reaction mixture was prepared by combining 90 μL of 20 mM Tris-HCl buffer (pH 7.5), 500 nM S-DNA, 20 μM Thioflavin T (ThT), and 10 μL of plasma. Subsequent fluorescence analysis was performed to quantify the ClO^−^ concentration in the samples. The study complied with the National Institutes of Health’s Guidelines (Publication No. 85-23, 1996 revision) and was approved by Heze University’s Ethics Committee. Animals were randomly allocated to control or SIH groups using a random number table generated by GraphPad Prism 9.0. No exclusion criteria were applied.

## 3. Results

### 3.1. Experimental Principle

**Figure 1:**
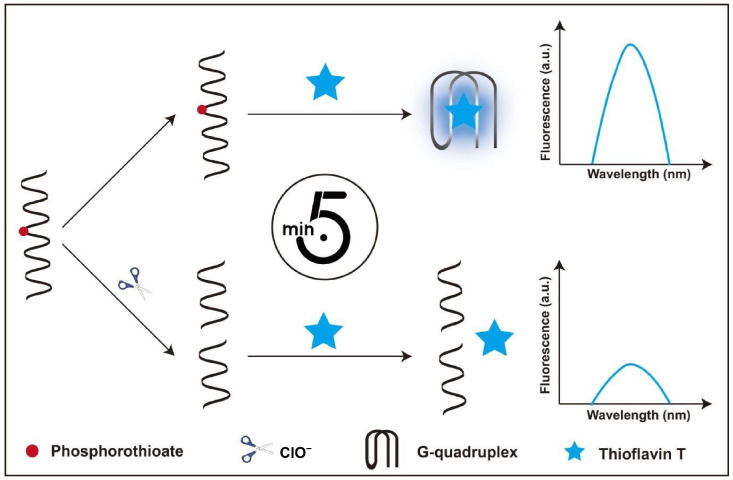
Schematic illustration of ClO^−^ detection. The reaction takes only 5 minutes.

Based on the specific cleavage of PS-modified DNA by ClO^−^ and the fluorescence enhancement of ThT upon binding to G-quadruplexes, a label-free fluorescent sensing platform for ClO^−^ was constructed (Figure 1). In the absence of ClO^−^, ThT binds to the G-quadruplex structure formed by the S-DNA, leading to a strong fluorescence signal. In the presence of ClO^−^, the PS linkage in the S-DNA undergoes hydrolysis, resulting in cleavage of the S-DNA into two fragments. This cleavage prevents the formation of G-quadruplex structure, thereby reducing the binding between ThT and the S-DNA and causing a significant decrease in fluorescence intensity at 490 nm. Thus, the concentration of ClO^−^ can be quantitatively determined according to the change in fluorescence intensity.

### 3.2. Feasibility Test of the Strategy

**Figure 2:**
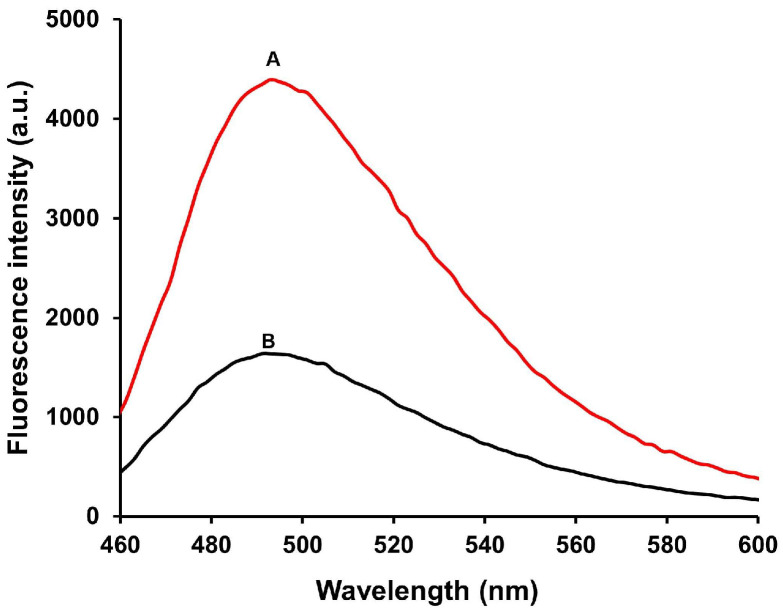
Fluorescence emission spectra in the absence (A) and the presence (B) of 50 μM ClO^−^.

The feasibility of the proposed method was verified by comparing the fluorescence spectra of the system in the absence and in the presence ClO^−^ (Figure 2). In the absence of ClO^−^ (curve A), the system exhibited a high fluorescence intensity, indicating the formation of the G-quadruplex/ThT complex. In the presence of 50 μM ClO^−^ (curve B), a significant reduction in fluorescence intensity was observed, confirming that ClO^−^ induces cleavage of the PS-modified S-DNA, which disrupts G-quadruplex formation. These results demonstrate that the proposed method is simple, effective, sensitive, and rapid for ClO^−^ detection.

### 3.3. Reaction Condition Optimization

**Figure 3:**
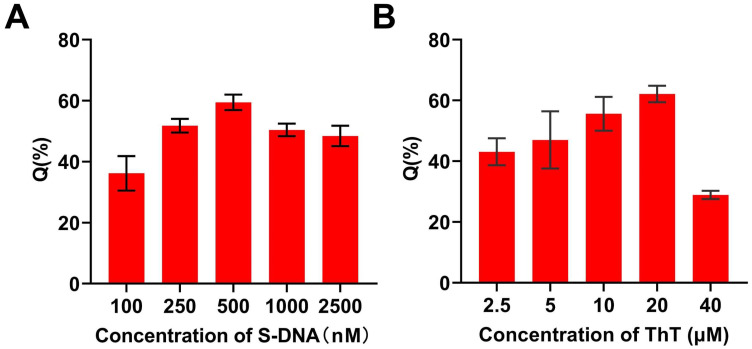
Optimization of the ClO^−^ detection conditions: (**A**) The concentration of S-DNA; (**B**) The concentration of ThT. Data represent the mean ± SD for three replicates.

To improve the detection sensitivity, the concentrations of S-DNA and ThT were optimized. As shown in Figure 3A, the fluorescence quenching efficiency (Q%) increased with increasing S-DNA concentration and reached a maximum at 500 nM. Further increases in S-DNA concentration did not significantly improve the Q%, so 500 nM was selected as the optimal S-DNA concentration. For ThT, the Q% increased with increasing concentration up to 20 μM (Figure 3B), after which it decreased. This decrease in Q% is attributed to an increased fluorescence background caused by the high concentration of ThT. Therefore, 20 μM was chosen as the optimal ThT concentration for subsequent experiments.

### 3.4. Quantitative Analysis of ClO^−^

**Figure 4:**
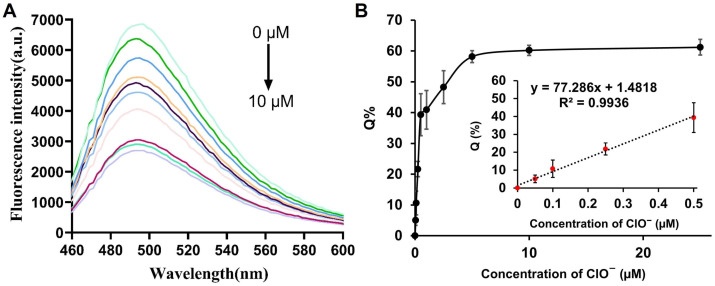
(**A**) Fluorescence emission spectra of sensing system in the presence of increasing amounts of ClO^−^ (0, 0.05, 0.1, 0.25, 0.5, 1, 2.5, 5, 10 and 25 μM). (**B**) The linear relationship between Q% and the concentration of ClO^−^. Inset: the linear relationship at low ClO^−^ concentration. Error bars show the standard deviation of three experiments.

Under the optimized conditions, the quantitative performance of the method for ClO^−^ detection was evaluated. As shown in Figure 4A, the fluorescence intensity at 490 nm gradually decreased with increasing ClO^−^ concentration in the range of 0–25 μM. The Q% exhibited a good linear relationship with ClO^−^ concentration (Figure 4B). Notably, at low ClO^−^ concentrations (0–0.5 μM), a linear correlation was observed between the fluorescence intensity and ClO^−^ concentration with a correlation coefficient (R^2^) of 0.9936 (inset of Figure 4B). Based on the calculation formula for the Limit of Detection (LOD), which is defined as 3.3 times the standard deviation (σ) divided by the slope (S) of the calibration curve, the detection limit of our method is 41.2 nM. The sensitivity of our assay was higher than that of other previously reported fluorescent ClO^−^ detection methods (Table 1) [21,23,24,25,26,27,28]. Furthermore, the total reaction time of our assay was 5 min, which was much shorter than that of fluorescent ClO^−^ detection methods.

### 3.5. Specific Analysis

**Figure 5:**
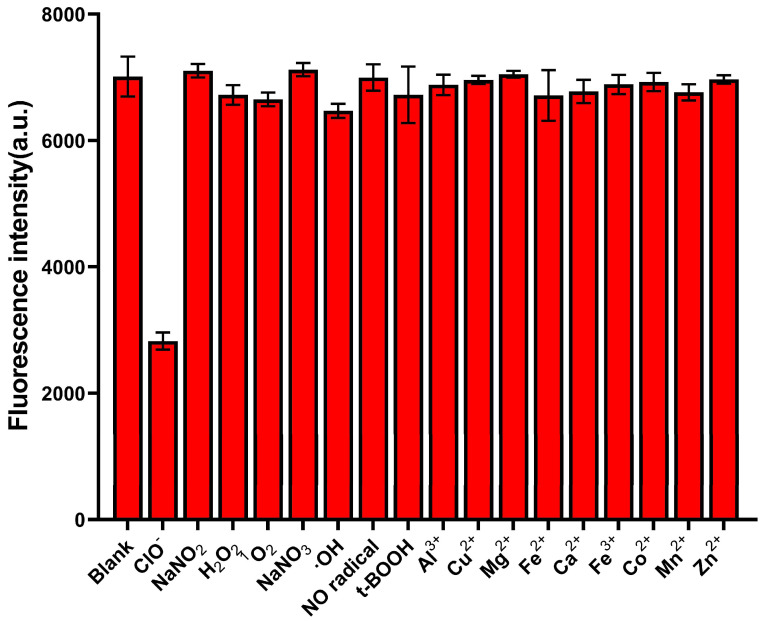
The fluorescence intensity of S-DNA (500 nM) after reacting with 25 μM ClO^−^ or 25 μM other ROS and metal ions.

The specificity of this method towards ClO^−^ was evaluated. Neither the other relevant ROS (25 μM NaNO_2_; H_2_O_2_; ^1^O_2_; NaNO_3_; ·OH; NO radical; t-BOOH) nor biologically relevant metal ions (25 μM Al^3+^; Cu^2+^; Mg^2+^; Fe^2+^; Ca^2+^; Fe^3+^; Co^2+^; Mn^2+^; Zn^2+^) led to significant fluorescence changes (Figure 5). By contrast, the fluorescence intensity decreased markedly after adding ClO^−^, indicating the excellent specificity of the method for ClO^−^.

### 3.6. Analysis of ClO^−^ in Human Cells

**Figure 6:**
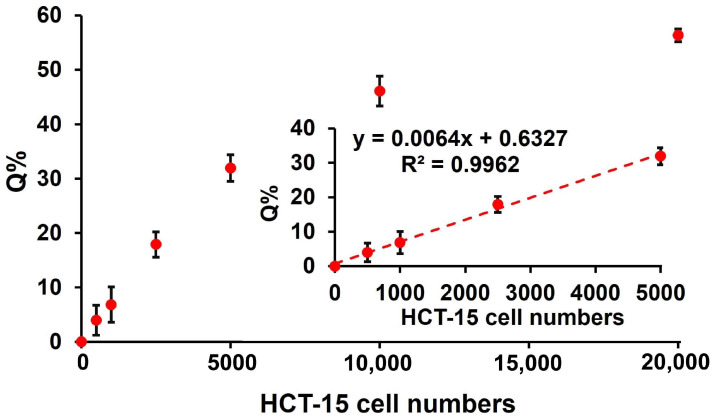
HCT-15 cell lysate analysis utilizing the procedures of the proposed method. The error bar reflects three separate measurements (mean ± SD).

To test the method’s applicability to human cells, we applied it to lysates of the colorectal cancer line HCT-15. The calibration curve was linear over the range of 0–5000 cells (Figure 6), with a regression equation of y = −0.4536x + 7058.7 and a correlation coefficient (R^2^) of 0.9963, where x represents the number of HCT-15 cells and y corresponds to the fluorescence intensity. These data indicate that the assay can reliably quantify ClO^−^ in human cells.

### 3.7. Investigation of ClO^−^ Levels in Plasma of Rats Exhibiting SIH

**Figure 7:**
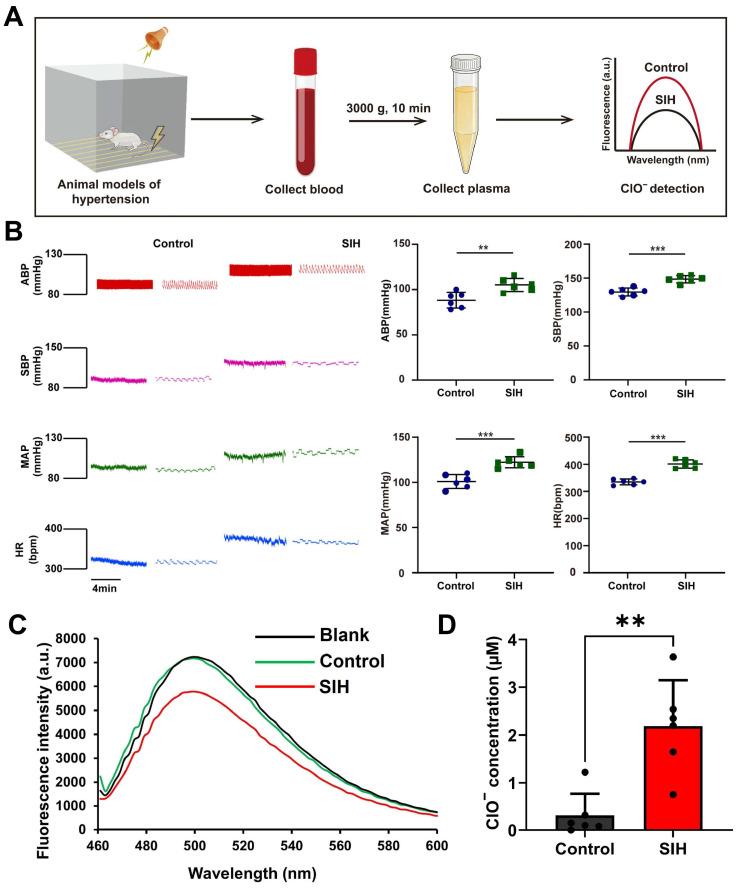
Evaluation of plasma ClO^−^ levels in a rat model of SIH. (**A**) Summary of experimental protocols in animals. (**B**) Analysis of cardiovascular parameters (ABP, SBP, MAP, HR). (**C**,**D**) FL imaging in the plasma illustrating differences between control and SIH rats. All statistical analyses were performed using GraphPad Prism 9.0. Quantitative data are presented as mean ± standard deviation (SD), with *n* = 6 independent biological replicates per group. Unpaired two-tailed Student’s *t*-test was used for comparisons between the control group and SIH group. (** *p* < 0.01, *** *p* < 0.001 vs. control).

SIH is characterized by sustained elevations in blood pressure due to chronic stress, which can lead to severe health complications if left untreated [29]. While accumulating evidence suggests that reactive oxygen species (ROS) in plasma exacerbate SIH, the specific role of ClO^−^ in this context remains largely unexplored. Therefore, this study aimed to quantify ClO^−^ levels in the plasma of SIH rats using our established methodology. Before analyzing the plasma samples, the matrix effect was systematically evaluated to ensure the accuracy of quantification. Specifically, known quantities of ClO^−^ were added to the plasma samples from healthy rats. As shown in Table 2, no significant matrix interference was observed for the detection of ClO^−^ in rat plasma. The recovery values and relative standard deviations (RSDs) further confirmed that the proposed method is sufficiently robust and reliable for the direct quantification of ClO^−^ in this complex biological matrix. Figure 7A illustrates the animal study design, which included six control rats and six rats with SIH as the experimental group, with a total of 12 animals used in this study. As illustrated in Figure 7B, SIH rats exhibited significantly elevated arterial blood pressure (ABP), systolic blood pressure (SBP), mean arterial pressure (MAP), and heart rate (HR) compared with control animals. Consistent with these physiological changes, SIH rats displayed notably lower fluorescence intensity than controls (Figure 7C). The authors analyzed a total of 12 real samples, including 6 blank plasma samples and 6 plasma samples from SIH model rats. The calibration curve was constructed using ClO^−^ at concentrations of 0, 0.05, 0.1, 0.25, and 0.5 μM under optimized conditions, and all experiments were performed under identical conditions. The ClO^−^ concentration in plasma samples was quantified by substituting the measured Q% value into the corresponding linear regression equation. Furthermore, as highlighted in Figure 7D, plasma ClO^−^ levels were substantially increased in the SIH group relative to controls, with statistical significance (*p* < 0.01). These findings suggest that ClO^−^ may serve as a diagnostic biomarker for SIH, highlighting the platform’s value in translating ROS biomarkers from preclinical research to clinical applications.

## 4. Conclusions

In summary, we have developed a highly sensitive method for ClO^−^ detection with a limit of detection (LOD) of 41.2 nM. The S-DNA requires no fluorophore labeling, offering a convenient and low-cost approach. Owing to the high specificity and rapidity of the hydrolysis reaction between PS and ClO^−^, the established method exhibits superior selectivity for ClO^−^ detection compared to other reported assays. Having been successfully applied to the quantitative determination of endogenous ClO^−^ in human cells and the plasma of SIH rats, this method holds substantial potential as a simple, low-cost tool for ClO^−^ analysis in clinical and physiological research, particularly for SIH-related diagnostic screening and pathogenesis studies. However, this study has notable limitations. This study exclusively used male Sprague-Dawley rats, with no exploration of gender differences in ClO^−^ metabolism or hypertension susceptibility, which limits the generalizability of the findings to female populations or other species. The sensor’s performance in more complex biological matrices (e.g., tissues) and in vivo scenarios remains untested, and the small sample size (*n* = 6 per group) may reduce statistical power for detecting subtle differences. Nevertheless, the sensor’s simplicity, low cost, and biological applicability still render it valuable for future ClO^−^ research, with room for optimization to address these constraints.

## Figures and Tables

**Table 1:** Comparison of different methods for hypochlorous acid detection.

Methods	Year	LOD (µM)	Linear Range (µM)	Signal Mode	Ref.
Phenothiazine probe	2023	0.472	0–30	Turn on	[23]
Cinnamic acid probe	2025	0.099	0–70	Turn on	[24]
2–aminoanthracene probe	2025	0.14	0.5–2.5	Turn off	[25]
PCH probe	2022	2.102	0–100	Turn on	[26]
HON1-CN	2025	0.48	0–50	Turn on	[27]
BOD−CN probe	2023	0.083	/	Turn on	[28]
Block DNA–G-Quadruplex/Thioflavin T	2024	0.01	0–1	Turn on	[21]
This work	2026	0.0412	0–0.5	Turn off	/

**Table 2:** Results for the detection of ClO^−^ in plasma.

ClO^−^ Spiked (µM)	ClO^−^ Found (µM)	Recovery (%)	RSD (%)
0.250	0.257 ± 0.015	102.67%	5.95
0.500	0.497 ± 0.021	99.33%	4.19

## Data Availability

Data presented in this study is contained within the article. Further inquiries can be directed to the corresponding authors.
